# Maternal caregiving moderates relations between maternal childhood maltreatment and infant cortisol regulation

**DOI:** 10.1111/jcpp.14171

**Published:** 2025-04-08

**Authors:** Miriam Chasson, Jennifer Khoury, Michelle Bosquet Enlow, Karlen Lyons‐Ruth

**Affiliations:** ^1^ Department of Psychiatry Cambridge Hospital, Harvard Medical School Boston Massachusetts USA; ^2^ Department of Psychology Mount Saint Vincent University Halifax Nova Scotia Canada; ^3^ Department of Psychiatry and Behavioral Sciences Boston Children's Hospital Boston Massachusetts USA; ^4^ Department of Psychiatry Harvard Medical School Boston Massachusetts USA

**Keywords:** Infancy, cortisol, intergenerational transmission, abuse, neglect, mother–infant interaction

## Abstract

**Background:**

Children of maltreated mothers are at increased risk for adverse physical and psychological health. Both prenatal and postnatal alterations in offspring biological stress systems have been proposed as mechanisms contributing to such transmission. The aim of the current study was to assess whether maternal postnatal care of the infant moderated any effect of maternal childhood maltreatment on infant cortisol output during a mild stressor at 4 months of age.

**Methods:**

Participants included 181 mother–infant dyads, screened at recruitment to result in 57.4% reporting one or more forms of childhood maltreatment. Mothers were assessed for quality of caregiving, and infants were assessed for infant salivary cortisol output during the Still‐Face Paradigm at infant age 4 months. Maternal childhood maltreatment was assessed using the Maltreatment and Abuse Chronology of Exposure (MACE) self‐report scales.

**Results:**

Greater severity of maternal childhood neglect interacted with higher levels of maternal disoriented caregiving to predict higher infant cortisol output over the course of the Still‐Face Paradigm. In contrast, maternal childhood abuse interacted with higher levels of maternal negative‐intrusion to predict lower infant cortisol output. Greater maternal role confusion was linked to greater infant cortisol output regardless of maternal maltreatment history.

**Conclusions:**

Maternal caregiving may moderate the effects of risk factors existing prior to the infant's birth. Disoriented caregiving in the context of maternal childhood neglect and negative‐intrusive behavior in the context of maternal childhood abuse were associated with opposite directions of effect on infant stress hormone output. The results suggest that interventions addressing risks from both prenatal and postnatal periods may be most effective in mitigating intergenerational effects of maltreatment.

## Introduction

Offspring of mothers who have experienced childhood maltreatment are at increased risk for adverse outcomes, including self‐regulation difficulties (Delker, Noll, Kim, & Fisher, 2014) and elevated rates of poor physical and psychological health (Plant, Pawlby, Pariante, & Jones, 2018; Racine, Plamondon, Madigan, McDonald, & Tough, 2018; Roberts et al., 2014; Zhang, Mersky, Gruber, & Kim, 2023). Fetal programming, or alterations in offspring biological stress systems during gestation, has been proposed as one mechanism underlying these intergenerational effects (Buss et al., 2017; Moog et al., 2018). Maternal stress hormones pass through the placenta during gestation and are hypothesized to shape developing infant stress response systems, including the hypothalamic–pituitary–adrenal (HPA) axis (Glover, O'Donnell, O'Connor, & Fisher, 2018; Moog et al., 2018).

As discussed by Bateson et al. (2004), prenatal programming can have both adaptive and maladaptive potential, depending on how closely the original environment of maternal adaptation forecast by her biological cues conforms to the infant's postnatal environment. Given that the accuracy of the “weather forecast” from the mother's cues is uncertain (Bateson et al., 2004), it would also be adaptive to allow postnatal cues to alter the prenatal ‘weather forecast’ to conform more closely to actual environmental conditions during the postnatal period. Evidence from controlled rodent and primate studies of prenatal stress has confirmed the presence of postnatal mechanisms that recalibrate infant biological systems to better conform to the postnatal environment (Francis, Szegda, Campbell, Martin, & Insel, 2003; Lemaire, Lamarque, Le Moal, Piazza, & Abrous, 2006; Maccari et al., 1995; Sanchez, 2006; Sapolsky, 1997; Wakshlak & Marta, 1990). Maternal postnatal caregiving has been shown to be one of these mechanisms (Del Cerro et al., 2010; Maccari et al., 1995). Some evidence of postnatal recalibration also exists among human infants, with negative outcomes associated with maternal gestational anxiety moderated by the security of mother–infant attachment (Bergman, Sarkar, Glover, & O'Connor, 2010), and negative outcomes associated with exposure to elevated maternal gestational cortisol moderated by quality of postnatal interaction (Nazzari, Fearon, Rice, Molteni, & Frigerio, 2022).

Consistent with the fetal programming hypothesis, some effects of maternal childhood maltreatment on infant development are evident at birth or shortly thereafter. For example, both lower gray matter volume (Moog et al., 2018) and increased connectivity between the amygdala and medial prefrontal cortex, an index of adversity‐accelerated development (Hendrix et al., 2021), have been reported among newborns of mothers with childhood maltreatment. Maternal childhood maltreatment has also been linked to alterations in neurophysiological regulation later in infancy, including both lower baseline cortisol (Brand et al., 2010) and sustained higher cortisol levels across reactivity and recovery periods after a stressor (Khoury, Beeney, Shiff, Bosquet Enlow, & Lyons‐Ruth, 2021). Additionally, reduced correspondence of cortisol levels between maltreated mothers and their infants has been repeatedly observed (Brand et al., 2010; Fuchs, Möhler, Resch, & Kaess, 2016; Khoury et al., 2021).

In the current study, infant cortisol responses were assessed in the context of the Still‐Face Paradigm (SFP; Tronick, Als, Adamson, Wise, & Brazelton, 1978), a standardized mild stressor. Although the SFP reliably elicits infant negative affect (Mesman, Van IJzendoorn, & Bakermans‐Kranenburg, 2009), lower‐risk infants often exhibit declining cortisol levels in the SFP (Feldman, Singer, & Zagoory, 2010; Grant et al., 2009; Khoury et al., 2021; Lewis & Ramsay, 2005; Martinez‐Torteya et al., 2014; Montirosso, Tronick, Morandi, Ciceri, & Borgatti, 2013), whereas higher‐risk infants tend to exhibit sustained higher cortisol levels across the procedure (Crockett, Holmes, Granger, & Lyons‐Ruth, 2013; Erickson et al., 2019; Feldman et al., 2010; Grant et al., 2009; Khoury et al., 2021; Lewis & Ramsay, 2005; Martinez‐Torteya et al., 2014; but see Broeks et al., 2021). Risk factors assessed have included less sensitive maternal interaction (Crockett et al., 2013; Erickson et al., 2019; Grant et al., 2009; Martinez‐Torteya et al., 2014), severity of maternal childhood maltreatment (Khoury et al., 2021), and the presence of maternal anxiety or depression (Grant et al., 2009; Martinez‐Torteya et al., 2014). In addition, the absence of maternal touch during play and reunion (Feldman et al., 2010), and greater sad, but not angry, affect (Lewis & Ramsay, 2005) were also associated with sustained elevated cortisol. Given that infant risk has been more strongly related to sustained higher cortisol levels across the SFP than to a specific pattern of reactivity and recovery to the stressor, infant total cortisol output was used as the primary outcome measure in the current study.

Maladaptive caregiving can also serve as a direct stressor for the infant. Controlled rodent studies, in particular, have shown that stress response networks are altered by poor maternal care in the early postnatal period (Champagne et al., 2008; Weaver & Meaney, 1997). The caregiving behaviors most reliably linked to increased glucocorticoid release involve low maternal nurturance or unpredictability (Baram et al., 2012; Drury, Sánchez, & Gonzalez, 2016; Turecki & Meaney, 2016).^1^ Low maternal nurturance/unpredictability is associated with a host of alterations in pup development that persist into adulthood, including changes in HPA axis regulation, amygdala circuitry, and behavioral functioning (e.g., Champagne et al., 2008; Drury et al., 2016; Turecki & Meaney, 2016). Disrupted care has also been linked to offspring HPA axis dysregulation among non‐human primates (e.g., McCormack et al., 2022; Sanchez, McCormack, & Howell, 2015).

Fewer studies have assessed whether postnatal caregiving interacts with prenatal adversity to influence infant HPA axis activity. Quality of postnatal care can moderate effects of prenatal stress on the glucocorticoid system among rodents (Maccari et al., 1995). Among human dyads, higher maternal cortisol in the third trimester of pregnancy was shown to interact with lower postnatal emotional availability to predict elevated infant cortisol reactivity to inoculation stress at 3 months (Nazzari et al., 2022).

Notably, research on *direct* (not intergenerational) effects of child maltreatment suggests that different child neurobiological outcomes may be associated with abuse versus neglect (McLaughlin, Weissman, & Bitrán, 2019). Among older children and adults, exposure to abuse has been associated with reduced volume of the right amygdala, the brain region central to threat detection and fear regulation (McLaughlin et al., 2019; Teicher, Samson, Anderson, & Ohashi, 2016), whereas neglect has been associated with reductions in cortical gray matter and deficits in cognitive function (Machlin et al., 2023; Schäfer et al., 2023). Whether direct experiences of abuse or neglect are associated with differential effects on the HPA axis is less clear, with overall allostatic load (sustained stress) thought to play a major role in whether cortisol response is elevated or blunted (McEwen, 2001).

Few studies have examined whether *the mother's* childhood abuse versus neglect has different intergenerational effects on the development of infant stress response systems. Consistent with McLaughlin et al. (2019), Lyons‐Ruth et al. (2023) found that maternal childhood abuse was associated with lower infant right amygdala volume and maternal childhood neglect with lower infant gray matter volume. These findings suggest that the intergenerational effects of maternal childhood abuse versus neglect on infants' developing HPA axis regulation may also be different. McLaughlin and colleagues (Cuartas, Weissman, Sheridan, Lengua, & McLaughlin, 2021) have further argued that experiences of threat (abuse) and deprivation (neglect) should each be viewed as continua, with less severe experiences having similar but graded effects (e.g., spanking would have less severe effects than physical abuse). This view advocates considering aversive caregiving on a continuum of threat (Cuartas et al., 2021) and disengaged caregiving on a continuum of deprivation (Doom et al., 2022). Given this literature, it is important to assess maternal caregiving with instruments that capture both more aversive and more disengaged caregiving.

The current study aimed to extend the literature by assessing how maternal childhood maltreatment and postnatal maternal care interrelate in influencing infant cortisol regulation. The first hypothesis was that maternal childhood abuse and maternal childhood neglect are both related to altered infant cortisol output during a mild laboratory stressor. Although previous cortisol studies have examined maternal childhood maltreatment as a single variable (e.g., Khoury et al., 2021; Martinez‐Torteya et al., 2014), it is important to establish whether both aspects of maternal childhood maltreatment are similarly related to altered infant cortisol output. The second hypothesis was that relations between maternal childhood abuse/neglect and infant cortisol output are moderated by the quality of postnatal caregiving, such that the association between maternal childhood abuse or neglect and infant cortisol output is potentiated in the context of poor‐quality postnatal care. This hypothesis was based on findings in both rodent and human studies reviewed above that gestational risk exposures can be moderated by the postnatal environment. Finally, exploratory analyses examined whether specific aspects of disrupted postnatal care are more strongly related to altered infant cortisol output. This analysis was exploratory because rodent studies suggest that low maternal nurturance/unpredictability is related to increased glucocorticoid response among pups and human studies indicate that insensitive maternal interaction is related to greater infant cortisol response, but neither literature has specifically differentiated between the effects of low nurturing and harsh/aversive components of insensitive maternal care.

## Method

### Participants

Mother–infant pairs (*N* = 181) were recruited into the Mother‐Infant Neurobiological Development (MIND) Study through prenatal classes, obstetric and pediatric clinics, community flyers, and local birth records. Potential participants were screened in an initial phone interview using the Adverse Childhood Experiences questionnaire (Felitti et al., 1998) to ensure that at least half (57.4%) of the mothers had experienced one or more forms of childhood maltreatment (physical, sexual, emotional abuse; witnessed domestic violence; physical, emotional neglect). Additional questions screened for the following exclusion criteria: (a) English not a primary language spoken at home, (b) maternal age over 44 years at the time of infant birth, (c) infant born before 36 weeks gestation and/or weighing <2,500 g, and (d) infant congenital disorder/condition. Racial/ethnic composition of the sample included 59% White, non‐Hispanic, 5% Hispanic, 7% Black, 1% Asian, and 29% multiracial/multiethnic.

### Ethical considerations

The study was approved by the Institutional Review Board [Partners Healthcare IRB Protocol #: 2014P002522]. Mothers provided informed consent prior to the initiation of study activities.

### Procedure

Assessments were conducted in the participant's homes by two master's level researchers. Home visits consisted of the videotaped SFP, collection of maternal and infant saliva samples, and completion of questionnaires. After obtaining informed consent, a baseline cortisol sample was taken from both mother and infant. Next, the infant was placed in an infant seat, and the mother sat opposite the infant. The mother was asked to follow the directions for the SFP (see Methods below). After the SFP ended, the mother filled out questionnaires while the infant either sat in her lap or played nearby. Additional cortisol samples were collected at +20 min and +40 min after the end of the 2‐min Still‐Face Period.

### Measures

#### Still‐Face Paradigm

Mothers and infants engaged in a 10‐min videorecorded Still Face Paradigm (SFP) (Tronick et al., 1978) at infant age 4 months. During the SFP, the mother was instructed to play with her infant normally for 3 min (Play Period), then to freeze her face of all emotion and not speak or interact with the infant for 2 min (Still‐Face Episode), then to resume normal face‐to‐face interaction with the infant for 5 min (Reunion Period; Tronick et al., 1978). Mothers were instructed not to use toys or other objects (e.g., cellphones) during the SFP, but they were allowed to touch their infants as part of play or soothing during the play and reunion episodes. The SFP is a validated mild stressor for infants, eliciting reduced positive and increased negative affect (Mesman et al., 2009).

#### Maternal disrupted interaction

Maternal behaviors were coded from video recordings of the reunion period of the SFP using the Atypical Maternal Behavior Instrument for Assessment and Classification (AMBIANCE; Lyons‐Ruth, Bronfman, & Parsons, 1999), by coders who were naïve to all other study data, including maternal maltreatment history. Because the Still‐Face period constitutes a mild stressor for the infant, the reunion period is considered the most revealing context for observing the caregiver's sensitivity in regulating infant arousal. In addition, in a separate sample, maternal interaction coded during the reunion episode was associated with elevated infant cortisol levels across the SFP (Crockett et al., 2013). Thus, on both theoretical and empirical grounds, the reunion period was chosen for coding maternal behavior.

Coders noted the severity and frequency of relevant behaviors in rating five dimensions of disrupted maternal interaction on 7‐point scales (1–7), with higher scores indicating greater disruption: (1) *affective communication errors* (contradictory affective signals to the infant, inappropriate or inadequate responses to the infant's cues); (2) *role/boundary confusion* (soliciting the infant's attention or affection to the self in ways that override or ignore the infant's signals); (3) *disorientation* (disoriented, odd, frenetic, or deferential behavior toward the infant, often characterized by odd affect, false affect, affect unrelated to the interaction, or affective disconnection); (4) *negative‐intrusive interaction* (harsh or critical verbal communication and/or physical behavior, such as mocking, teasing, or poking the infant); and (5) *withdrawal* (creating physical or emotional distance from the infant, such as leaning away from the infant, averting gaze, sitting silently). Additional specific behavioral examples relevant to each AMBIANCE dimension are shown in Table 1. Interrater reliability between two coders on 30 randomly selected videos was strong: disorientation ICC = .93, withdrawal ICC = .80, negative‐intrusive interaction ICC = .88, affective communication errors ICC = .94, role confusion ICC = .90. AMBIANCE ratings of overall disrupted interaction are stable over periods from 6 months to 7 years (Madigan et al., 2006) and predict maladaptive outcomes from infancy through adolescence (Lyons‐Ruth, Bureau, Holmes, Easterbrooks, & Brooks, 2013; Madigan et al., 2006; Yarger, Bronfman, Carlson, & Dozier, 2020).

**Table 1:** Atypical Maternal Behavior Instrument for Assessment and Classification (AMBIANCE): Coding system dimensions, subdimensions, and behavioral examples

Dimension 1: Affective Communication Errors	Subdimension 1A: Contradictory signaling to child Sweet voice with derogatory, demanding, or impatient message. Uses friendly tone while maintaining threatening posture
	Subdimension 1B: Failure to initiate responsive behavior to infant's cues: Does not attempt to soothe infant when distressed. Does not respond to clear infant cue directed to the caregiver
	Subdimension 1C: Inappropriate responding to infant's cues: Mother smiles when infant is angry, upset, afraid, or sad. Attempts to minimize or discount infant's display of distress
Dimension 2: Role/Boundary Confusion	Subdimension 2A: Role confusion: Demands show of affection from infant. Seeks attention from infant while infant is looking away. Demands the infant entertain parent with cute behaviors
	Subdimension 2B: Treats child as sexual/spousal partner: Speaks in hushed intimate tones to infant
Dimension 3: Fearful/Disorientation	Subdimension 3A: Fearful behavior: Appears frightened, apprehensive, or deferential in relation to infant. Handles infant in a timid or helpless manner
	Subdimension 3B: Disorientation or dissociative behavior: Deadened or flattened affect leaving empty feel to interaction. Exhibits rapid shifts in affect unrelated to environment. Shifts frenetically from topic to topic or activity to activity
	Subdimension 3C: Fearful or disoriented voices: Exhibits ‘ghost‐like’ whispering. Exhibits stilted voice that seems affectively disconnected
Dimension 4: Negative‐Intrusiveness	Subdimension 4A: Physical communications: Touches infant in a manner which appears to be affectionate but is irritating to the infant. Physically crowds or hovers closely to infant
	Subdimension 4B: Verbal communications: Laughs at infant. Makes negative comment about infant
	Subdimension 4C: Inappropriately attributes negative feelings or motivation to infant: Suggests negative motivation to innocuous behaviors. Personalizes infant's behavior as negative
	Subdimension 4D: Exerts control using objects: Removes toy from infant despite engagement (include pacifier, parent or infant fingers/toes). Withholds toy from infant (include pacifier, parent or infant fingers/toes)
Dimension 5: Withdrawing Behavior	Subdimension 5A: Creates physical distance from infant: Averts gaze. Adopts a posture designed to keep infant at a distance (e.g., sits at a distance, leans away, turns away)
	Subdimension 5B: Use of verbal communication to maintain distance: Does not greet infant after a separation (e.g., still‐face episode). Interacts silently with infant
	Subdimension 5C: Directs away from self to other aspects of environment: Directs infant away from self by using pacifier or child's finger. Redirects infant to other aspects of environment as an apparent substitute for closer engagement or contact with parent

Table based on AMBIANCE coding manual (Bronfman et al., 2021). For a complete listing of all 147 behaviors included as indicators of each AMBIANCE Dimension and Subdimension (see Haltigan et al., 2019).

#### Infant cortisol output

Infant saliva samples were collected three times during the SFP: before starting interaction (baseline), 20 min after the still‐face period ended (+20 min), and 40 min after the still‐face period ended (+40 min). The SFP was conducted at home to mitigate the impact of travel and an unfamiliar laboratory environment on infant cortisol levels. The SFP was conducted in the afternoon (average time = 14:00; *SD* 1 hr and 38 min) to avoid the confounding effects of diurnal decline in the morning. Mothers were instructed not to allow their infants to eat or drink for 30 min prior to the first saliva collection to minimize contamination. Sorbette swabs (Salimetrics, State College, PA) were used to collect saliva samples. Samples were stored at −20°C. Saliva samples were sent to the Kirschbaum laboratory at the Technical University of Dresden for cortisol extraction. Samples were centrifuged for 15 min at 3000 rpm prior to extraction. Cortisol was assayed in duplicate using chemiluminescence immunoassay kits with high sensitivity (IBL International, Hamburg, Germany). Average inter‐ and intra‐assay variation was <10%. Area under the curve with respect to ground (AUCg) was calculated to index total cortisol output over the course of the SFP (Pruessner, Kirschbaum, Meinlschmid, & Hellhammer, 2003).

#### Maternal childhood maltreatment

Mothers completed the 75‐item Maltreatment and Abuse Chronology of Exposure self‐report scales (MACE; Teicher & Parigger, 2015). The MACE assesses the severity of childhood maltreatment, including 10‐point scales for verbal abuse, non‐verbal emotional abuse, physical abuse, sexual abuse, emotional neglect, and physical neglect. The four abuse scales were summed to create an abuse severity index (possible range 0–40), and the two neglect subscales were summed to create a neglect severity index (possible range 0–20). MACE scale scores correlate highly with other measures of childhood maltreatment and have high test–retest reliability (Teicher & Parigger, 2015).

#### Sociodemographic variables

Sociodemographic variables, assessed by maternal interview, included infant age, gestational age, and sex, maternal age and education, and annual family income.

### Data analytic strategy

IBM SPSS Statistics 29 was used for preliminary statistical analyses to check the normality of distributions and to identify covariates. Due to skewness and kurtosis, cortisol values (baseline, +20 and +40) were log10 transformed and winsorized to reduce the effect of outliers; then AUCg was computed. Potential covariates were included in regression models if they were related to the dependent variable. Regression models were analyzed using the lavaan package in R (Rosseel, 2012), using maximum likelihood estimation with FIML to handle any missing data and non‐normal distributions. Missing data included 12.7% of cortisol samples at one or more of the three time points needed to compute AUCg and 1.1% of the maternal behavior data. Little's MCAR test indicated that both maternal and infant data [*χ* ^2^ (13) = 9.194, *p* = .98] were missing at random, making them suitable for analysis using FIML. Results with 95% confidence intervals that do not include zero are considered statistically significant at *p* < .05. Multiple independent tests related to a specific aim were adjusted using the Benjamini‐Hochberg method to control the false discovery rate, with a significance threshold of .05 (Benjamini & Hochberg, 1995). Moderation regression models were run to assess the interaction between maternal childhood abuse or neglect and maternal caregiving on infant AUCg. If aspects of caregiving did not moderate the effects of maternal childhood abuse or neglect, interaction terms were removed and main effects models were evaluated.^2^

## Results

### Descriptive statistics and covariate analyses

Descriptive statistics for major study variables appear in Table 2. As can be seen from the raw means for infant cortisol levels at each time point over the SFP, infant cortisol levels in the sample declined from baseline.^3^ None of the potential covariates (cortisol collection time, infant age, gestational age and sex, maternal age and education, family income) were associated with infant AUCg (*r*'s range = −.09 to .07, all *p* ns).

**Table 2:** Sociodemographic characteristics and descriptive statistics

	*M* (*SD*)/% (*N*)	Range
Maternal age	*M =* 32.06 (*SD =* 4.25)	20–44
Maternal education		
Less than high school degree	1.1% (*n* = 2)	–
High school	15.6% (*n* = 28)	–
Associate degree	7.8% (*n* = 14)	–
Bachelor's degree	23.9% (*n* = 43)	–
Master's degree	35.0% (*n* = 63)	–
Doctoral degree	16.7% (*n* = 30)	
Annual family income		
$0 to $15,000	8.8% (*n* = 16)	–
$16,000 to $25,000	6.1% (*n* = 11)	–
$26,000 to $50,000	14.9% (*n* = 27)	–
$51,000 to $75,000	13.3% (*n* = 24)	–
$76,000 to $100,000	16.6% (*n* = 30)	–
$101,000 to $150,000	24.3% (*n* = 44)	–
$151,000+	16.0% (*n* = 29)	–
Gestational age at birth (weeks)^a^	*M =* 39.62 (*SD =* 1.43)	36–42
Infant age in days	*M =* 144.47 (*SD =* 30.69)	95–259
Infant sex		
Female	53.3% (*n* = 96)	–
Male	46.7% (*n* = 84)	–
MACE neglect severity	*M* = 2.71 (*SD* = 3.86)	0–16
MACE abuse severity	*M* = 10.10 (*SD* = 8.66)	0–35
Affective communication errors	*M* = 3.84 (*SD* = 1.71)	1–7
Role confusion	*M* = 3.00 (*SD* = 1.47)	1–7
Disorientation	*M* = 3.10 (*SD* = 1.65)	1–7
Negative intrusion	*M* = 4.122 (*SD* = 1.49)	1–7
Withdrawal	*M* = 2.04 (*SD* = 1.45)	1–7
Saliva collection time (minutes post midnight)	*M* = 824.12 (*SD* = 98.60)	672–1,110
Baseline cortisol^b^	*M =* 5.899 (*SD* = 8.30)	0.44–66.57
Post 20‐min cortisol^b^	*M =* 4.733 (*SD* = 8.48)	0.50–69.81
Post 40‐min cortisol^b^	*M =* 5.237 (*SD =* 10.73)	0.45–79.95
Total infant cortisol output (AUCg) (Log transformed and winsorized)	*M* = 45.95 (*SD* = 4.71)	40.80–67.00

AUCg, area under the curve with respect to ground (total cortisol output); MACE, Maltreatment and Abuse Chronology of Exposure scale.

^a^ Infants born <36 weeks gestation were not eligible for the study.

^b^ Data are presented as raw values, with three outliers removed, using a 3 *SD* threshold to identify outliers. Cortisol metric is nmol/L.

Bivariate relations among the main study variables are presented in Table 3. Table 3 indicates that mother's childhood neglect was associated with her disorientation and role confusion, while mother's childhood abuse was not significantly associated with maternal behavior. Infant cortisol output was associated only with maternal role confusion. Neither mother's childhood abuse nor neglect was associated with infant cortisol output in the bivariate correlations.

**Table 3:** Associations among aspects of maternal disrupted interaction, types of maternal childhood maltreatment, and infant total cortisol output (AUCg)

Variable	1	2	3	4	5	6	7	8
1. MCNeglect	–							
2. MCAbuse	.643***	–						
3. Disorientation	.157*	.076	–					
4. Withdrawal	−.053	.010	.292***	–				
5. Negative‐intrusion	.142	.119	.407***	−.023	–			
6. Role confusion	.173*	.104	.409***	−.178*	.507***	–		
7. Affect. comm. errors	.059	−.007	.571***	.250***	.614***	.325***	–	
8. Infant AUCg	.114	−.044	.132	.062	.065	.199*	.091	–

Affect. comm. errors, affective communication errors; AUCg, area under the curve with respect to ground, or total cortisol output; MCAbuse, severity of maternal childhood abuse; MCNeglect, severity of maternal childhood neglect.

*p < .05, **p < .01, ***p < .01.

### Maternal childhood abuse and neglect and infant cortisol output

**Figure 1:**
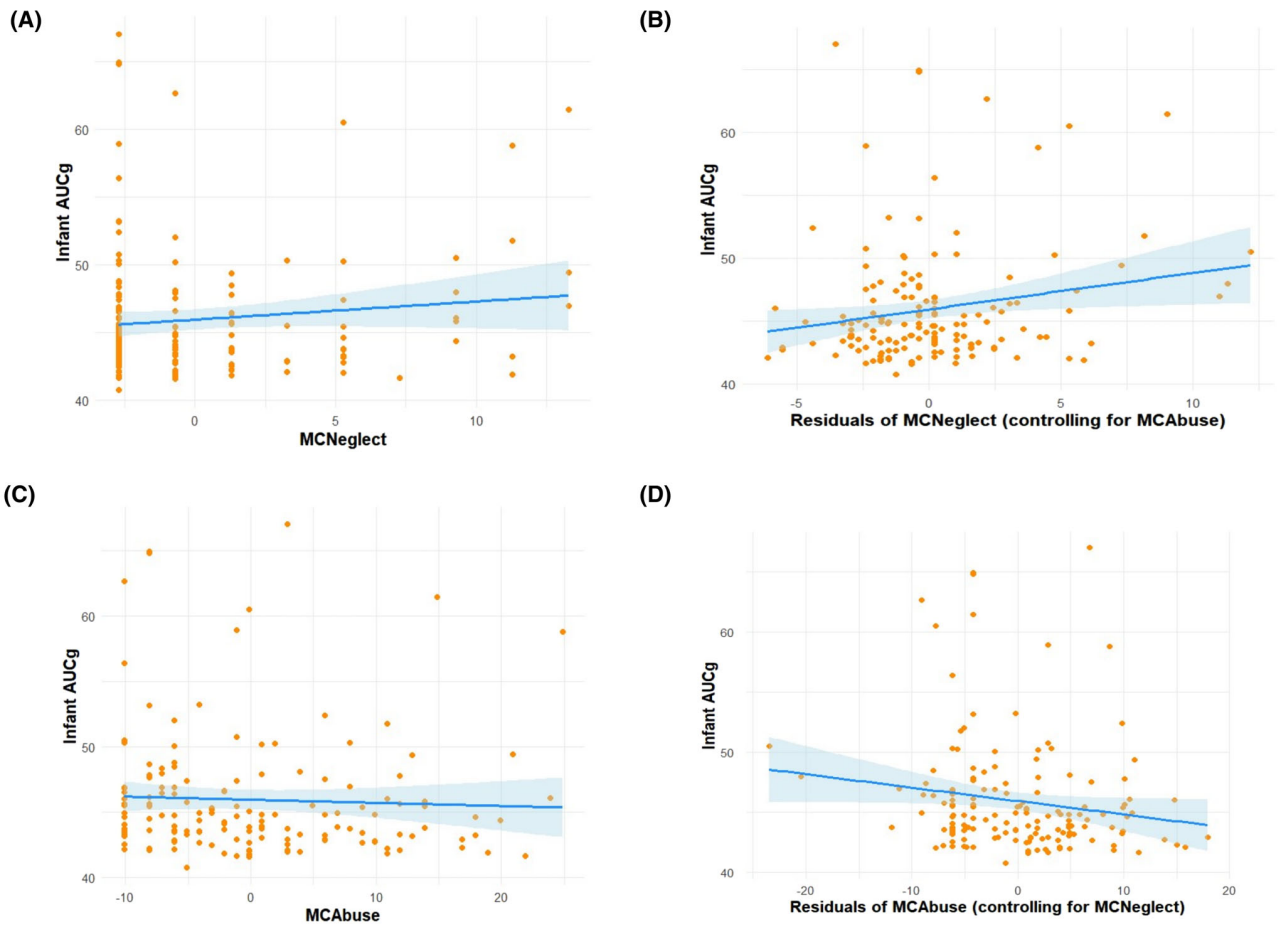
Distribution of infant cortisol levels (AUCg) as a function of the severity of maternal childhood neglect, (A) without and (B) with the severity of maternal childhood abuse controlled; and distribution of infant cortisol levels (AUCg) as a function of the severity of maternal childhood abuse, (C) without and (D) with the severity of maternal childhood neglect controlled. MCAbuse, maternal childhood abuse; MCNeglect, maternal childhood neglect; Plots show standardized residuals from regression models. AUCg, area under the curve with respect to ground. AUCg metric is nmol/L. Regression data presented with estimation of missing data using FIML. Plotted data reflect *N* = 181

**Figure 2:**
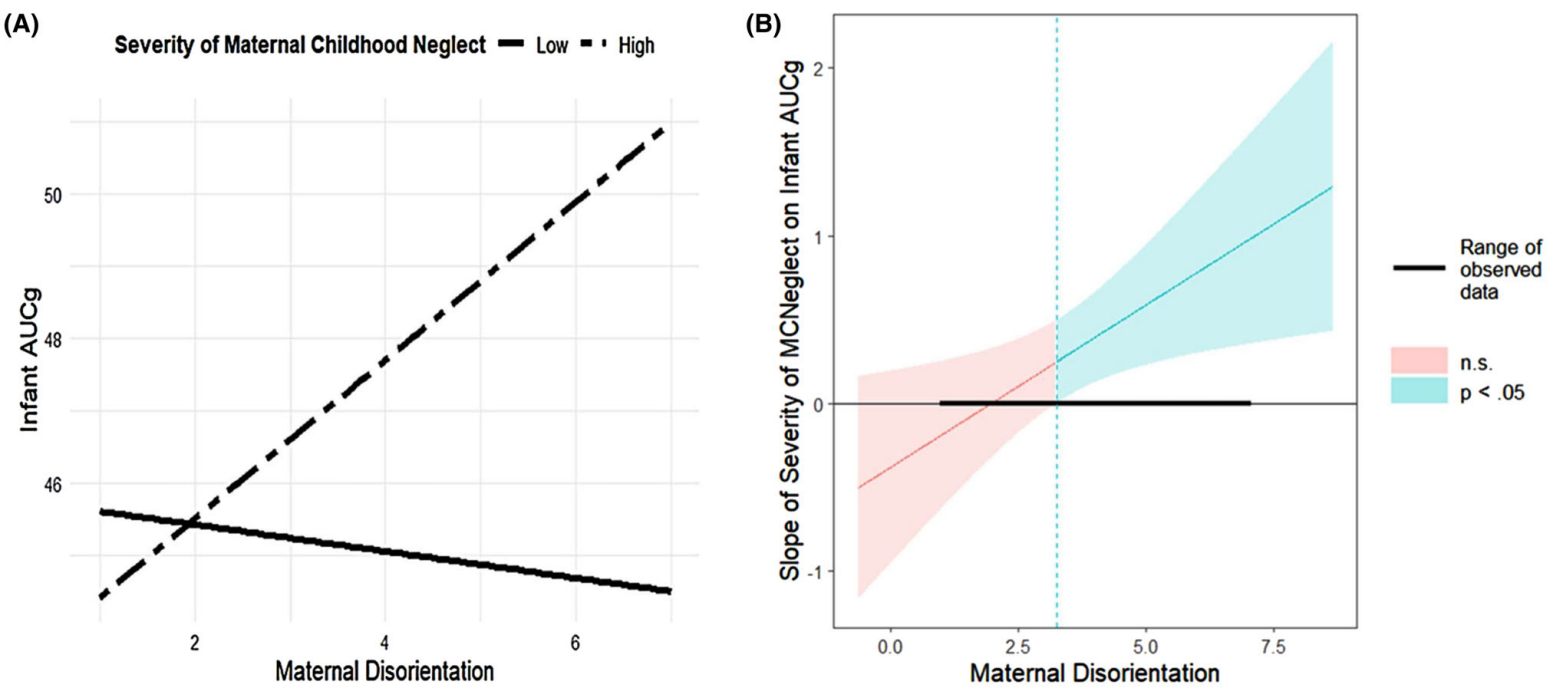
Graphic illustration and region of significance of the interaction between severity of maternal childhood neglect and maternal disorientation on infant cortisol output (AUCg). Figure (A) plots the association between maternal disorientation and infant cortisol output (AUCg) for two levels of maternal childhood neglect: low (solid line) and high (dashed line). Low neglect is defined as 1 *SD* below the mean; high neglect is defined as 1 *SD* above the mean. AUCg, area under the curve with respect to ground (total cortisol output). (B) Region of significance plotted using the Johnson–Neyman method (Johnson & Neyman, 1936). Graph derived using linear regression without FIML (*N* = 152). MCNeglect, maternal childhood neglect. The range of observed values of maternal disorientation is 1.00, 7.00. When maternal disorientation is outside the interval [−4.04, 3.20], the slope of maternal neglect on infant AUCg is *p* < .05. Thus, maternal childhood neglect is significantly associated with higher infant AUCg when maternal disorientation is >3.20

Preliminary regression analyses assessed whether maternal childhood abuse and neglect should be entered into the same or separate regression models. Given the high correlation between the severity of neglect and the severity of abuse, *r* (*N* = 179) = .643, *p* < .001, multicollinearity assessment was conducted on the model including both abuse and neglect using the Variance Inflation Factor (VIF) and Tolerance methods. VIF and Tolerance values were 1.741 and 0.574, respectively. These metrics did not indicate multicollinearity concerns, as the VIF value was well below the accepted threshold of 5, and the Tolerance value exceeded the minimum threshold of 0.1. Consistent with bivariate correlations, when entered into separate regression models, neither maternal childhood abuse nor neglect was associated with infant AUCg (Table 4 and Figure 1A–1C). However, when included in the same regression model, both maternal childhood abuse and neglect were associated with infant AUCg, but in opposite directions (Table 4 and Figures 1B and 2D). Thus, the positive association of infant AUCg with maternal childhood neglect (*β* = 0.237, *SE* = 0.133, *p* = .030, 95% CI [0.027, 0.550]) was obscured when failing to control for the negative association of infant AUCg with maternal childhood abuse (*β* = −0.202, *SE* = 0.050, *p* = .029, 95% CI [−0.209, −0.011]), and vice versa. Therefore, subsequent regression analyses included both maternal childhood abuse and neglect in the same models.

**Table 4:** Regression coefficients from regression models with maternal childhood neglect and maternal childhood abuse as independent variables and infant total cortisol output (AUCg) as the dependent variable

Regression models on infant AUCg		*β*	*SE*	*Z*	95% CI	*p*	VIF^a^	Tolerance
1. Maternal childhood neglect	MCNeglect	0.109	0.115	1.151	−0.093, 0.358	.250		
2. Maternal childhood abuse	MCAbuse	−0.043	0.048	−0.492	−0.117, 0.070	.623		
3. Maternal childhood neglect and abuse	MCNeglect	0.237	0.133	2.165	0.027, 0.550	.030	1.741	0.574
	MCAbuse	−0.202	0.050	−2.180	−0.209, −0.011	.029	1.741	0.574

*N* = 181. AUCg, area under the curve with respect to ground (total cortisol output); MCAbuse, maternal childhood abuse; MCNeglect, maternal childhood neglect.

^a^ Given the high correlation observed between maternal childhood neglect and abuse, multicollinearity diagnostics were conducted using Variance Inflation Factor (VIF) and Tolerance methods. Both metrics indicate no multicollinearity concerns, as the VIF values are well below the commonly accepted threshold of 5, and the Tolerance values exceed the minimum threshold of 0.1.

### Maternal childhood abuse and neglect and maternal disrupted interaction in relation to infant cortisol output

Separate moderation models were tested for each of the five aspects of maternal disrupted behavior in interaction with maternal childhood abuse and maternal childhood neglect. Moderation effects were significant for two of the models. Higher levels of maternal disorientation potentiated the effect of higher levels of maternal childhood neglect in relation to elevated infant cortisol output (*β* = 0.646, *SE* = 0.080, *p* = .016, 95% CI [0.035, 0.347], Table 5 and Figure 2A). Analysis of the region of significance for maternal childhood neglect by maternal disorientation demonstrated that maternal childhood neglect was associated with higher infant AUCg when the level of maternal disorientation was >3.20 (Figure 2B). No other aspect of maternal interaction moderated the association of maternal childhood neglect with infant AUCg (Table 5).

**Table 5:** Regression coefficients from moderation models examining the prediction of infant total cortisol output (AUCg) from the interactions of maternal childhood neglect and abuse with the five aspects of disrupted maternal interaction

Regression models on infant AUCg		*β*	*SE*	*Z*	95% CI	*p*	Adj. *p* value^a^
1. Disorientation	MCNeglect	−0.302	0.250	−1.470	−0.859, 0.123	.142	
	MCAbuse	0.111	0.098	0.616	−0.131, 0.252	.538	
	Disorientation	0.120	0.322	1.057	−0.291, 0.973	.291	
	MCNeglect*Disorientation	0.646	0.080	2.398	0.035,0.347	.016	.032
	MCAbuse*Disorientation	−0.423	0.028	−1.869	−0.108, 0.003	.062	.062
2. Withdrawal	MCNeglect	0.359	0.265	1.647	−0.083, 0.958	.100	
	MCAbuse	−0.138	0.101	−0.742	−0.274, 0.123	.458	
	Withdrawal	0.182	0.472	1.247	−0.337, 1.515	.212	
	MCNeglect*Withdrawal	−0.162	0.177	−0.510	−0.437, 0.257	.610	.810
	MCAbuse*Withdrawal	−0.071	0.055	−0.240	−0.120, 0.094	.810	.810
3. Negative‐intrusion	MCNeglect	−0.061	0.434	−0.170	−0.925, 0.777	.865	
	MCAbuse	0.380	0.124	1.667	−0.036, 0.451	.096	
	Negative intrusion	0.254	0.397	2.020	0.024, 1.579	.043	
	MCNeglect*Negative intrusion	0.351	0.108	0.844	−0.120, 0.302	.399	.399
	MCAbuse* Negative intrusion	−0.731	0.030	−2.559	−0.137, −0.018	.010	.020
4. Role confusion	MCNeglect	−0.046	0.216	−0.257	−0.480, 0.368	.797	
	MCAbuse	−0.115	0.085	−0.737	−0.229, 0.104	.461	
	Role confusion	0.131	0.393	1.063	−0.352, 1.189	.288	
	MCNeglect*Role confusion	0.295	0.065	1.347	−0.040, 0.215	.178	.356
	MCAbuse*Role confusion	−0.091	0.023	−0.511	−0.057, 0.034	.609	.609
5. Affective errors	MCNeglect	−0.007	0.273	−0.030	−0.543, 0.527	.976	
	MCAbuse	0.208	0.131	0.867	−0.143, 0.370	.386	
	Affective errors	0.185	0.339	1.499	−0.156, 1.171	.134	
	MCNeglect*Affective errors	0.282	0.078	0.991	−0.075, 0.230	.321	.321
	MCAbuse*Affective errors	−0.486	0.032	−1.818	−0.120, 0.005	.069	.138

*N* = 181. Main effects in the table are adjusted for interaction terms, so that main effects are not interpreted when interaction terms are included. Significant interaction terms survived correction, as shown. AUCg, area under the curve with respect to ground (total cortisol output); MCAbuse, maternal childhood abuse; MCNeglect, maternal childhood neglect.

^a^ Because five independent models were run, the 10 interaction terms of interest were adjusted to control the false discovery rate, with a significance threshold of .05, using the Benjamini and Hochberg (1995) procedure.

**Figure 3:**
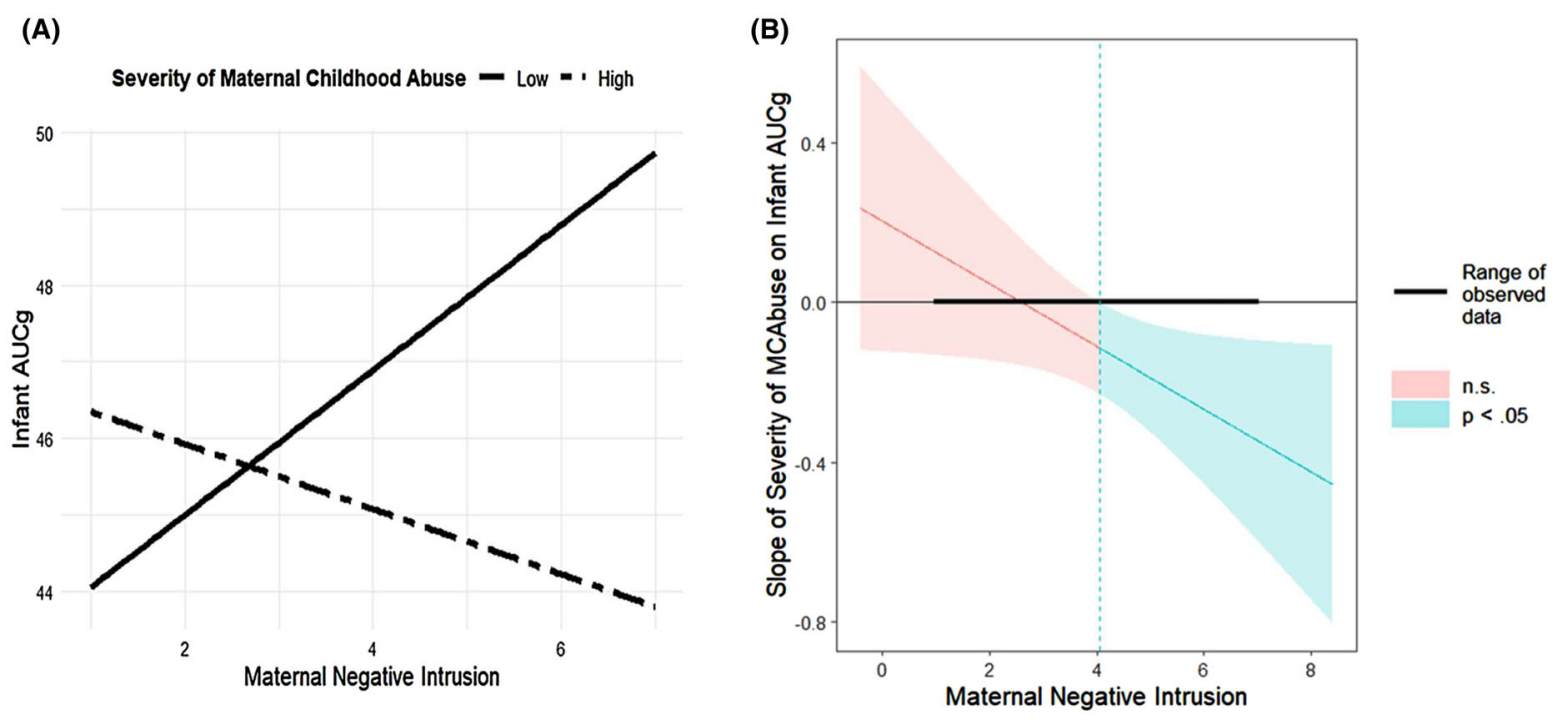
Graphic illustration and region of significance of the interaction between severity of maternal childhood abuse and maternal negative intrusion on infant cortisol output (AUCg). Figure (A) plots the association between maternal negative intrusion and infant cortisol output (AUCg) for two levels of maternal childhood abuse: low (solid line), and high (dashed line). Low abuse is defined as 1 *SD* below the mean; high abuse is defined as 1 *SD* above the mean. AUCg, area under the curve with respect to ground (total cortisol output). (B) Region of significance plotted using the Johnson–Neyman method (Johnson & Neyman, 1936). Graph derived using linear regression without FIML (*N* = 152). MCAbuse, maternal childhood abuse. The range of observed values of maternal negative intrusion is 1.00, 7.00. When maternal negative intrusion is outside the interval [−19.67, 4.12], the slope of maternal abuse on infant AUCg is *p* < .05. Thus, maternal childhood abuse is significantly associated with lower infant AUCg when maternal negative intrusion is >4.12

In addition, maternal negative‐intrusive interaction potentiated the *negative* association of maternal childhood abuse with infant AUCg, such that *lower* infant AUCg was observed in the dual context of higher maternal childhood abuse and higher negative‐intrusive interaction (*β* = −0.731, *SE* = 0.030, *p* = .010, 95% CI [−0.137, −0.018], Table 5 and Figure 3A). Analysis of the region of significance for this interaction indicated that higher levels of maternal childhood abuse were associated with lower infant AUCg when the level of maternal negative‐intrusive behaviors was >4.12 (Figure 3B). No other aspect of maternal interaction moderated the negative association of abuse with infant AUCg (Table 5).

Given that 10 moderation effects of interest were tested across the five independent models, adjusted *p*‐values for those 10 effects were also computed. The interaction of disorientation with maternal childhood neglect on higher infant AUCg remained significant (*p* = .032; Table 5), as did the interaction of negative‐intrusive interaction with maternal childhood abuse on lower infant AUCg (*p* = .020; Table 5).

Other aspects of maternal disrupted interaction, namely withdrawal, role confusion, and affective communication errors, did not interact with the mother's childhood maltreatment to predict infant cortisol. Therefore, main effects of these three aspects of maternal behavior on infant AUCg were assessed in a single regression model. Consistent with bivariate correlations in Table 3, role confusion was significantly associated with higher AUCg (*β* = 0.218, *SE* = 0.268, *p* = .010, 95% CI [0.169, 1.221]), while maternal withdrawal (*β* = 0.099, *SE* = 0.357, *p* = .367, 95% CI [−0.377, 1.021]) and maternal affective communication errors (*β* = −0.006, *SE* = 0.273, *p* = .948, 95% CI [−0.552, 0.517]) were not related to infant cortisol. Because these effects were tested within a single model, *p*‐values were already adjusted for the number of parameters in the model.

## Discussion

The current study examined the interplay between maternal childhood maltreatment and postnatal maternal caregiving in their relations to infant stress regulation in the first year of life. The major finding was that alterations in infant cortisol output were associated with specific interactions between aspects of maternal care and aspects of maternal childhood maltreatment. In finding that postnatal care potentiated the effect of risk factors present during the prenatal period, our results converge with those of Nazzari et al. (2022). They found that maternal emotional unavailability potentiated the effect of maternal gestational cortisol to predict elevated infant cortisol reactivity. In both the Nazzari et al. (2022) study and the present study, a different infant cortisol response was seen in the context of the joint combination of a maternal risk factor prior to infant birth (maternal childhood maltreatment or gestational cortisol) and maladaptive postnatal caregiving.

In addition, in the present study, different aspects of maternal behavior interacted with maternal childhood abuse versus neglect in relation to infant cortisol output. More disoriented care interacted with maternal childhood neglect to predict *higher* levels of infant cortisol output over the course of the stressor, and more negative‐intrusive behavior interacted with maternal childhood abuse to predict *lower* levels of infant cortisol output. In previous work, maternal disorientation has been associated with more neglect‐related outcomes and environments, including infant disinhibited social engagement behavior (e.g., indiscriminate approach to strangers; Lyons‐Ruth, Bureau, Riley, & Atlas‐Corbett, 2009; Lyons‐Ruth, Riley, Patrick, & Hobson, 2019), which is most often observed among infants in neglecting institutional care (Zeanah & Gleason, 2015). In the current sample, maternal disorientation was elevated among mothers who had experienced both childhood neglect and abuse compared to those experiencing abuse alone (Khoury et al., 2022). Thus, evidence to date suggests that maternal disorientation may be more strongly associated with neglecting environments. As such, disorientation may serve as one signal of maternal neglect to the infant stress response system.

Also importantly, among the 57 infants with MRI data in the current sample, maternal disorientation was indirectly related to enlarged infant amygdala volume through its relation to higher infant AUCg (Khoury et al., 2023). Notably, in that subsample, disorientation levels of 4 or more (out of a possible 7) were associated with greater infant cortisol output and, indirectly, to enlarged amygdala volume. Thus, the current finding that similar disorientation levels interacted with maternal childhood neglect to predict greater infant cortisol output also has implications for the development of stress‐sensitive brain regions.

Descriptively, maternal disorientation is defined by indices of emotional disconnection/affective distortion (odd affect, false affect, frenetic interaction; Lyons‐Ruth et al., 1999). In the early months of life, the exchange of mutually contingent and sensitive affective signals is essential to infant cortisol regulation (Feldman, 2003; Hostinar, Sullivan, & Gunnar, 2014). Notably, the dimension of maternal behavior associated with elevated infant cortisol was the dimension indexing disconnected (odd or false) maternal affect/behavior that blocks effective communication between mother and infant. Although differences in maternal behavior across species make comparisons difficult, disoriented maternal behavior may be consistent with rodent models of low maternal nurturance/maternal unpredictability, where the rodent mother is either disinclined or anxious and distracted from nurturing her infant pups (Drury et al., 2016; Turecki & Meaney, 2016). Rodent studies show that low maternal nurturance/maternal unpredictability is causally related to elevated offspring glucocorticoid levels and altered amygdala function (Champagne et al., 2008; Drury et al., 2016).

In contrast to these findings associated with maternal childhood neglect, the joint effect of maternal childhood abuse and negative‐intrusive caregiving was associated with *lower* infant cortisol output. Mothers who have experienced childhood abuse (Khoury et al., 2022), as well as parents who are currently abusing their children (Wilson, Rack, Shi, & Norris, 2008), are more likely to display hostile and intrusive behavior with their offspring. The current results suggest that negative‐intrusive maternal behavior may potentiate an infant gestational vulnerability related to the mother's childhood abuse that results in lower infant cortisol output. Region of significance analyses indicated that levels of negative‐intrusion >4.12 were associated with lower infant cortisol output in the context of maternal childhood abuse. Because a rating of 5 (out of a possible 7) on this dimension indexes serious disruption in interaction (Bronfman, Madigan, & Lyons‐Ruth, 2021), scores above 4 would indicate notable negativity in mother–infant interaction. Among infants of non‐abused mothers, this negativity was associated with elevated infant cortisol (Figure 3A), consistent with other literature (Blair, Granger, Willoughby, & Kivlighan, 2006; Blair et al., 2008, 2015; Braren, Perry, Ursache, & Blair, 2019; Martinez‐Torteya et al., 2014). In contrast, among infants of abused mothers, there was no cortisol elevation to maternal negative‐intrusive behavior. The apparent suppression of cortisol seen in relation to maternal negativity among infants of abused mothers could indicate an early specific blunting of the infant's stress regulation system to cues associated with abuse, increasing vulnerability to stress‐related disorders later in life (Fisher, Burraston, & Pears, 2005; Gunnar, 2020; Koss, Mliner, Donzella, & Gunnar, 2016).

This specificity in the aspects of maternal care that potentiate specific types of maternal childhood maltreatment suggests that intergenerationally transmitted prenatal vulnerabilities may be experience‐expectant. That is, the type of maternal childhood maltreatment may confer specific epigenetic (Scorza et al., 2020) or gestationally‐based (Buss et al., 2017; Moog et al., 2022) infant susceptibilities that are activated by corresponding features of the postnatal environment. Such need for redundancy in prenatal and postnatal environments would build resilience into the infant system, such that neither prenatal nor early postnatal adverse environments would be sufficient in themselves to alter the infant's stress physiology. Only when the postnatal environment ‘confirms’ the prenatal vulnerabilities associated with the mother's childhood adversity would the infant stress system be significantly affected. Given the novelty of the pattern of results reported here, additional study in diverse samples is warranted.

Another important aspect of the results is that maternal childhood abuse and neglect had opposite associations with infant cortisol, with abuse associated with lower and neglect with higher cortisol output. These opposing effects have important methodological implications. If neglect is not controlled when assessing effects of abuse and vice versa, the opposing effects may not be revealed or null findings may result. Current results point to the importance of including both types of maltreatment in the same model to assess potential differential relations to infant stress physiology.

Potential opposing effects of maternal childhood abuse and neglect on infant stress regulation are also important substantively. In controlled rodent studies, low maternal nurturance/unpredictability was associated with alterations in the stress physiology of rodent pups, including increased glucocorticoid output, changes in circuitry of the amygdala and hippocampus, and alterations in behavioral functioning that persist into adulthood (Drury et al., 2016; Turecki & Meaney, 2016). Thus, the current finding that neglect‐related aspects of maternal history and current behavior are related to increased cortisol response in early infancy is consistent with the body of rodent studies (Lyons‐Ruth, Chasson, Khoury, & Ahtam, 2024).

Lower activation of the infant HPA axis among infants of abused mothers has not previously been reported. While a large maltreatment literature on older children and adults indicates that HPA axis hypo‐responsivity can be associated with more sustained abuse, this hypo‐responsivity has been conceptualized as a later‐appearing adaptation to continued stress (McEwen, 2001). The current results suggest that hypo‐responsivity may be an earlier adaptation observed in infancy in the joint context of a maternal history of childhood abuse and current maternal negative‐intrusive interaction. However, many questions remain regarding the mechanisms that may contribute to blunted cortisol in early infancy, before sustained exposure to stress or maltreatment has occurred. Thus, there is still much to understand about how abuse and neglect experiences, both as directly experienced and as intergenerationally transmitted, may be related to early cortisol hyper‐ and hypo‐responsiveness.

Maternal role confusion was the only aspect of care to show a main effect on infant cortisol, with greater role confusion related to higher infant cortisol output. Maternal role confusion is characterized by the mother asking for attention or affection from the infant, as when the mother prompts the infant to talk to her or give her a kiss, or by the mother engaging in self‐referential talk to the infant, e.g., “Mommy's having a hard day.” Thus, at higher levels, role confusion is an index of abdication of a parental role, placing the infant in the role of attending to the parent's needs. Both Khoury et al. (2022) in the current sample and Guyon‐Harris, Madigan, Bronfman, Romero, and Huth‐Bocks (2021) in a separate sample found that mother's childhood neglect was associated with maternal role confusion in infancy (also see Table 3). Therefore, the higher infant cortisol associated with maternal role confusion appears consistent with the general finding that maternal neglect‐related indices are associated with higher infant cortisol output, whereas maternal abuse‐related indices are not. More extended consideration of why cues to neglect, but not abuse, might be associated with a stress response in early infancy can be seen in Lyons‐Ruth et al. (2024).

## Limitations and future directions

Findings should be interpreted in the context of study limitations. First, the study relied on a single stress‐inducing procedure (the Still‐Face Paradigm) to assess infant cortisol output. Because different stressors may elicit different stress responses, future research should assess infant cortisol in a variety of stress‐inducing situations. Second, retrospective self‐reporting of maternal childhood maltreatment could introduce recall errors, affecting the accuracy of the maltreatment indices (e.g., Reuben et al., 2016). Third, analyses were correlational, with all variables assessed at a single time point. Thus, the causal direction of effects cannot be determined. In addition, while the current sample included a range of income and educational levels, a sizeable percentage had higher socioeconomic status. Thus, results may not generalize to lower SES populations. Finally, although this study captures early differences in the infant stress response, it does not track how stress responses might change over time or relate to other aspects of infant brain and behavioral development. Longitudinal studies are needed to explore the implications of these results for later developmental outcomes.

## Conclusions

This study highlights the potential interaction between maternal childhood adversity and maternal postnatal care in influencing infant cortisol regulation. Notably, maternal childhood neglect interacted with disoriented caregiving to predict elevated infant cortisol output to a stressor, whereas maternal childhood abuse interacted with negative‐intrusive caregiving behaviors to predict lower infant cortisol output. These findings suggest that the specific nature of both maternal childhood adversity and quality of caregiving must be considered together to fully understand the development of infant stress physiology. Interventions that target maltreated mothers prenatally and continue into the postnatal period may be most beneficial in altering the intergenerational transmission of adversity.

## Ethical considerations

The current study was approved by the Partners Healthcare Institutional Review Board [IRB Protocol #: 2014P002522] and parents provided written informed consent.

**Key points**

- Maternal childhood maltreatment is associated with adverse child outcomes, including disruptions in cortisol regulation.
- Postnatal care is associated with differences in offspring stress responses in rodent and human studies.
- No studies to date have assessed how postnatal care interacts with maternal childhood maltreatment to influence infant cortisol regulation.
- Results indicated that maternal childhood abuse and neglect had different relations to infant cortisol output, in interaction with specific aspects of maternal care.
- Maternal childhood adversity and postnatal care need to be considered together to understand mechanisms that shape infant stress responses.
- Interventions that target maltreated mothers prenatally and continue into the postnatal period may be most beneficial in preventing the intergenerational transmission of adversity.

## Supporting information


**Table S1.** Standardized coefficients and confidence intervals from separate mediation models with maternal childhood abuse or neglect as the independent variable and infant cortisol output as the dependent variable, controlling for severity of the other form of maternal childhood maltreatment.
**Figure S1.** Mean cortisol levels across three time points (Baseline, +20 m, +40 m) for the full sample.

## Data Availability

The data from the authors' lab referred to in this paper are available from the senior author upon reasonable request.

## Endnotes

1 Different laboratories use different terms for the anxious and fragmented care provided by stressed dams or dams bred for low nurturing behavior.

2 Based on precedent in rodent studies, the primary study hypothesis was that postnatal factors would interact to moderate or potentiate prenatal risk. However, to assess whether mediational models provided a better fit for the data, complete results of mediation analyses are presented in the Table S1. These analyses find no evidence for a mediational model.

3 Results of a repeated measures ANOVA on the three infant cortisol levels, as well as a graphic plot of the log‐transformed and winsorized infant cortisol values can be seen in the Figure S1.
