# Supplementary material for: Maternal caregiving moderates relations between maternal childhood maltreatment and infant cortisol regulation
Source: J Child Psychol Psychiatry. 2025 Apr 8;66(11):1627–41. doi: 10.1111/jcpp.14171 (PMC12571947; doi:10.1111/jcpp.14171)
Supplement: Supplementary file 1 — Table S1. Standardized coefficients and confidence intervals from separate mediation models with maternal childhood abuse or neglect as the independent variable and infant cortisol output as the dependent variable, controlling for severity of the other form of maternal childhood maltreatment. Figure S1. Mean cortisol levels across three time points (Baseline, +20 m, +40 m) for the full sample. [file JCPP-66-1627-s001.docx]

**Supplement**

**Results**

**Supplementary Mediation Models.** The primary study hypothesis was that postnatal factors would moderate prenatal risk, as reported in the main text. However, to assess whether mediational models provided a better fit for the data, mediation analyses are also reported here. Mediation analyses assessed whether maternal childhood neglect or abuse were associated with infant cortisol output via maternal caregiving quality, with maternal childhood abuse controlled in analyses of maternal childhood neglect and vice versa. No significant indirect associations of maternal childhood neglect or maternal childhood abuse with infant cortisol output were observed through any of the five caregiving variables assessed (Table S1). However, consistent with findings in the main text, direct associations of maternal childhood neglect and maternal childhood abuse with infant cortisol output were observed across all models (Table S1).

**Analysis of the Overall Sample Cortisol Trajectory across the Still-Face Paradigm.** The literature indicates that, although the Still-Face Paradigm (SFP) reliably elicits negative affect from infants, it does not reliably produce an elevated cortisol response following the 2-minute still-face episode. In the current sample, a repeated measures ANOVA revealed a significant main effect of time, *F*(2, 314) = 5.204, *p* = .006, indicating that cortisol levels varied across the three time points of the SFP: baseline (prior to start of the procedure), +20 minutes after the end of the still-face episode, and +40 minutes after the end of the still-face episode. Specifically, cortisol levels were elevated at baseline, dropped sharply by the reactivity phase (+20 minutes), and showed a slight increase during the recovery phase (+40 minutes), without returning to baseline levels. These findings suggest that, although there is significant variation in cortisol levels over the procedure, the overall trajectory for the sample as a group reflects a decrease in cortisol from baseline through recovery, rather than an increase following the still-face episode, as would be consistent with a classic stress reactivity response.

**Table S1.** *Standardized coefficients and confidence intervals from separate mediation models with maternal childhood abuse or neglect as the independent variable and infant cortisol output as the dependent variable, controlling for severity of the other form of maternal childhood maltreatment*

| **Model** | **Effect Type** | ***β*** | ***SE*** | ***Z*** | **95% CI** | ***P*** |
| --- | --- | --- | --- | --- | --- | --- |
| MCNeglect^1^ → Disorientation → Infant AUCg | Indirect Effect | 0.021 | 0.022 | 1.162 | -0.018, 0.070 | 0.245 |
|  | Direct Effect | 0.219 | 0.127 | 2.102 | 0.018, 0.517 | 0.036 |
| MCNeglect → Withdrawal → Infant AUCg | Indirect Effect | -0.008 | 0.016 | -0.638 | -0.041, 0.021 | 0.523 |
|  | Direct Effect | 0.246 | 0.130 | 2.309 | 0.045, 0.555 | 0.021 |
| MCNeglect → Negative-Intrusion → Infant AUCg | Indirect Effect | 0.007 | 0.012 | 0.654 | -0.016, 0.032 | 0.513 |
|  | Direct Effect | 0.232 | 0.131 | 2.157 | 0.026, 0.539 | 0.031 |
| MCNeglect → Role Confusion → Infant AUCg | Indirect Effect | 0.033 | 0.030 | 1.337 | -0.019, 0.100 | 0.181 |
|  | Direct Effect | 0.207 | 0.126 | 2.007 | 0.006, 0.500 | 0.045 |
| MCNeglect → Affective Errors→ Infant AUCg | Indirect Effect | 0.009 | 0.016 | 0.642 | -0.021, 0.042 | 0.521 |
|  | Direct Effect | 0.230 | 0.130 | 2.154 | 0.025, 0.535 | 0.031 |
| MCAbuse^2^ → Disorientation→ Infant AUCg | Indirect Effect | -0.005 | -0.003 | 0.007 | -0.016, 0.011 | 0.690 |
|  | Direct Effect | -0.201 | -0.109 | 0.051 | -0.208, -0.010 | 0.030 |
| MCAbuse → Withdrawal → Infant AUCg | Indirect Effect | 0.006 | 0.006 | 0.562 | -0.008, 0.015 | 0.574 |
|  | Direct Effect | -0.209 | 0.05 | -2.263 | -0.212, -0.015 | 0.024 |
| MCAbuse → Negative-Intrusion→ Infant AUCg | Indirect Effect | 0.003 | 0.004 | 0.378 | -0.006, 0.009 | 0.706 |
|  | Direct Effect | -0.204 | 0.052 | -2.160 | -0.212, -0.010 | 0.031 |
| MCAbuse → Role Confusion→ Infant AUCg | Indirect Effect | -0.002 | 0.011 | -0.117 | -0.022, 0.020 | 0.907 |
|  | Direct Effect | -0.202 | 0.05 | -2.210 | -0.207, -0.012 | 0.027 |
| MCAbuse → Affective Errors → Infant AUCg | Indirect Effect | -0.006 | 0.006 | -0.572 | -0.015, 0.008 | 0.568 |
|  | Direct Effect | -0.198 | 0.049 | -2.190 | -0.204, -0.011 | 0.029 |

**Note.** *N* = 181. ^1^MCNeglect = maternal childhood neglect; ^2^MCAbuse = maternal childhood abuse; CIs that do not contain zero are significant at *p* < .05; all models controlled for the other form of maternal childhood maltreatment. As can be seen, none of the indirect effects are significant. Direct effects of maternal childhood neglect on infant AUCg are significant and positive, consistent with the main manuscript; Direct effects of maternal childhood abuse on infant AUCg are significant and negative, consistent with the main manuscript.


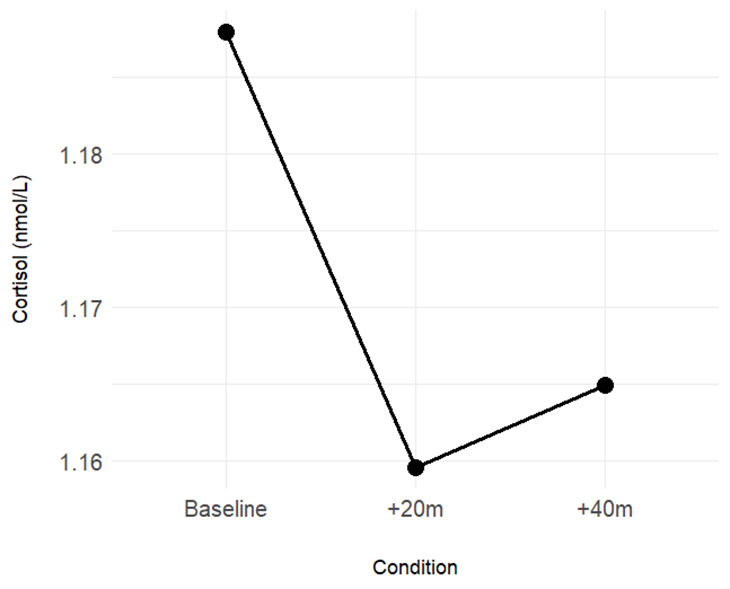


**Figure S1.** *Mean cortisol levels across three-time points (Baseline, +20m, +40m) for the full sample*
**Note:** This figure represents cortisol means for each timepoint, using log transformed and winsorized values, across the entire sample. Due to missing data for some cortisol assays, *N* = 170 for baseline, 166 for +20m and 169 for +40m.
